# Waist-to-height ratio and its associations with body mass index in a sample of Tuscan children in primary school

**DOI:** 10.1186/s13052-017-0372-x

**Published:** 2017-06-07

**Authors:** Francesca Santomauro, Chiara Lorini, Francesca Pieralli, Giuditta Niccolai, Paola Picciolli, Stefania Vezzosi, Guglielmo Bonaccorsi

**Affiliations:** 1 Department of Health Science, University of Florence, Viale G.B. Morgagni, 48, 50134 Florence, Italy; 2 School of Specialization in Hygiene and Preventive Medicine, University of Florence, Viale G.B. Morgagni, 48, 50134 Florence, Italy; 3 Local Health Unit Toscana Centro, Viale Matteotti, 19, 51100 Pistoia, Italy

**Keywords:** Waist-to-height ratio, Percentiles, Body mass index, Waist circumference, Children

## Abstract

**Background:**

Visceral obesity in children increases the risk of developing cardiovascular diseases. To evaluate overweight children, in addition to Body Mass Index (BMI), waist-to-height ratio (WHtR) can be used to predict cardiometabolic risk. The goal of this study is to describe WHtR in a sample of Tuscan children.

**Methods:**

A sample of children living in the province of Pistoia, Tuscany, was measured for the following anthropometric parameters: weight, height, and waist circumference. BMI and WHtR were calculated. For the latter indicator, a threshold of 0.5 was considered as a cardiovascular risk predictor. The subjects were classified into underweight, normal weight, overweight, and obese using Cole’s cut-offs.

**Results:**

The number of children enrolled were 1575 (821 males; 754 females), aged 6–11 years. Of them, 64.3% were normal weight, 4.9% underweight, 22.3% overweight, and 8.5% obese. Moreover, 12.8% had a WHtR ≥0.5 (85.7% males; 88.7% females). The average WHtR value was 0.45 ± 0.045, and was significantly different as per gender (F = 0.45 vs. M = 0.46). WHtR was significantly correlated with BMI (*r* = 0.766).

**Conclusion:**

The average WHtR value was in line with previous studies conducted among children of similar age groups. Large-scale perspective studies are needed to validate the Italian WHtR cut-offs for children.

## Background

Cardiometabolic risk factors are more prevalent among overweight or obese children and young adults than among people with healthy weight [1]; moreover, childhood overweight and obesity are increasingly becoming a public health issue [2]. Worldwide, the prevalence of overweight and obesity among children is more than 10% [3], and in Europe, childhood overweight is growing at an annual rate of about 400,000 cases [4].

A national survey conducted in 2014 by the Ministry of Health together with the National Institute of Health and the National Centre for Epidemiology, Surveillance, and Health Promotion on a representative sample of children attending primary school (8–9 years) showed a high national prevalence of overweight and obesity: 20.9% overweight and 9.8% obese [5].

To prevent overweight and obesity among adolescents and adults, there is need for early detection in young children. Several measures are used to detect obesity and the risk of obesity-related comorbidities. Body mass index (BMI) is the most commonly used measure for all ages, but supplementary measures such as waist circumference (WC) and waist-to-height ratio (WHtR) are specific indexes of abdominal fat. They have been proposed as markers of adiposity-related morbidity in children [6, 7].

WHtR may have an advantage over BMI, which does not provide any information about body fat distribution, in particular abdominal fat [8]. In fact, central obesity in children has been associated with the risk of cardiovascular and metabolic diseases [9], and poses greater health risks than total body fat [10] and peripheral distribution [11]. In adults, WHtR is found to be a better measure than BMI and WC as a predictor of obesity-related cardiometabolic risk factors. In children, on the other hand, it is less clear whether WHtR is better than BMI or WC in predicting obesity-related cardiometabolic risk [8]. Nevertheless, different studies state that WHtR is a good cardiometabolic risk indicator in children and adolescents [12–16]: it is simple and fast and can be used on a large scale to explain the metabolic consequences of obesity and identify abdominal obesity, particularly in individuals who would not be classified as overweight or obese by BMI [17]. Many authors have proposed a single WHtR cut-off value of 0.5, irrespective of age, sex, or ethnicity, as a valid predictor of higher cardiometabolic risk [6, 14, 18–20], although for obese children, a study has proposed a cut-off value of 0.6 [21].

The aims of this study were: 1) to describe the distribution of WHtR in a sample of six to 11-year-old children; and 2) to assess the association between WHtR and age, gender, and BMI in this age group.

## Methods

### Study population

This research was conducted in 2011–2013 in public primary schools in the province of Pistoia, Tuscany, comprising 20 municipalities. All the municipalities were asked to participate in this study, and four of them, with all of their 17 public primary schools (there are no private schools in these municipalities), agreed voluntarily.

Pupils (1912 children in total) aged 6–11 years were invited and recruited through written advertisements. On the day of the survey, written informed consent was obtained from the parents of 1575 children. About 6% of children (115) was absent on the day of measurement, and the written consent could not be obtained for 222 children. Information on the refusals was not collected.

With regard to ethnicity, about 10% of the children included in the study had parents of non-Italian origin; anyway, all the participants were Caucasians, and so data were managed as a whole, without stratification by ethnicity.

Anthropometric parameters were collected for each enrolled child.

The study was conducted according to the Declaration of Helsinki of the World Medical Association.

### Anthropometric measurements

The staffs were trained to collect all anthropometric measurements by following standardized international procedures [22]. For each subject in the sample, the following anthropometric variables were collected: weight, height, and waist circumference (WC). Weight was measured by using a mechanical balance scale (Seka Vogel and Halke, Hamburg, Germany) with a precision of 0.5 kg. Each child was measured shoeless, wearing underclothes and a T-shirt, about 2 hours after having consumed breakfast. Height was measured to a precision of 0.5 cm in the upright position using a portable stadiometer (SECA stadiometer). The stadiometer was checked for accuracy and the scale was calibrated before examination by using a reference metallic insertion tape. WC was measured midway between the lower rib margin and the superior anterior iliac spine at the end of normal expiration while the child was standing, by means of a non-stretchable tape. WC and height were detected by two trained operators.

WHtR was calculated as WC (cm) divided by height (cm). BMI was calculated as the ratio of weight (kg) to height (metres) squared. Underweight, overweight, and obesity were assessed by using the reference BMI cut-offs of Cole et al. [23].

### Statistical analysis

The values for the 3rd, 5th, 10th, 25th, 50th, 75th, 90th, 95th, and 97th percentiles for WHtR were calculated for boys and girls of all ages in one-year steps.

The descriptive analysis of anthropometric values was performed by gender and age. The Student’s t-test for independent data or analysis of variance (ANOVA) was applied to compare the mean values of anthropometric data. Chi2-test was applied to assess the associations between categorical data. Pearson correlation was used to measure the association between BMI and WHtR. For all the performed analyses, a *p* value of 0.05 was considered as significant.

Considering a WHtR cut-off value of 0.5, the children were classified into two categories—WHtR ≤0.5 and WHtR >0.5.

Statistical analyses were performed with IBM SPSS Statistics 23 software.

## Results

The final sample consisted of 1575 children, with 821 boys (52.1%) and 754 girls (47.9%), aged six to 11 years.

The age- and gender-specific mean (±SD) height, weight, BMI, WC, and WHtR are presented in Table 1.

**Table 1:** Anthropometric characteristics of the sample of six–11-year-old Tuscan children

	*N*	Height (m)^b,c^	Weight (kg) ^b,c^	BMI (kg/m^2^) ^b,c^	Waist circumference (cm) ^b,c^	Waist-to-height ratio^c^
Boys age (y)						
6	124	1.21 ± 0.05	24.13 ± 4.10	16.36 ± 2.04	55.71 ± 4.61	0.46 ± 0.03
7	169	1.28 ± 0.05^a^	28.87 ± 5.57^a^	17.60 ± 2.60	59.28 ± 5.77^a^	0.46 ± 0.04^a^
8	170	1.33 ± 0.06	31.60 ± 7.52	17.76 ± 3.12	60.78 ± 7.32^a^	0.46 ± 0.05
9	168	1.38 ± 0.06	35.10 ± 7.30	18.33 ± 2.97	62.51 ± 7.11	0.45 ± 0.04
10	148	1.44 ± 0.07	39.90 ± 8.76	19.14 ± 2.95	65.16 ± 7.60^a^	0.45 ± 0.04^a^
11	42	1.47 ± 0.07	43.54 ± 8.63	20.12 ± 3.29^a^	68.14 ± 7.24^a^	0.46 ± 0.05^a^
**All**	**821**	**1.34 ± 0.10**	**32.73 ± 8.93**	**18.00 ± 2.97**	**61.23 ± 7.43** ^**a**^	**0.46 ± 0.04** ^**a**^
Girls age (y)						
6	130	1.20 ± 0.06	24.76 ± 5.58	16.93 ± 2.63	56.03 ± 6.56	0.46 ± 0.05
7	144	1.26 ± 0.06^a^	27.49 ± 5.42^a^	17.17 ± 2.65	56.98 ± 5.71^a^	0.45 ± 0.04^a^
8	160	1.31 ± 0.06	31.05 ± 6.65	17.79 ± 2.94	59.08 ± 7.09^a^	0.45 ± 0.05
9	135	1.37 ± 0.06	35.01 ± 7.80	18.56 ± 3.20	61.84 ± 7.14	0.45 ± 0.05
10	135	1.43 ± 0.06	38.77 ± 8.19	18.72 ± 3.20	62.82 ± 6.89^a^	0.44 ± 0.04^a^
11	50	1.48 ± 0.07	41.04 ± 9.18	18.64 ± 3.10^a^	62.76 ± 7.51^a^	0.42 ± 0.04^a^
**All**	**754**	**1.32 ± 0.10**	**32.04 ± 8.77**	**17.88 ± 3.02**	**59.56 ± 7.24** ^**a**^	**0.45 ± 0.05** ^**a**^

^a^Boys vs. girls by age group: Student’s t-test for independent data (1574 d.f.) *p* < 0.05; ^b^among boys by age class: ANOVA (820 d.f.) *p* < 0.05; ^c^among girls by age group: ANOVA (753 d.f.) *p* < 0.05

The BMI values significantly increased with age among both genders, with more variability in boys than girls. The WHtR values significantly decreased with age in girls, with mean values varying between 0.46 and 0.42. With regard to gender variability within the same age group, statistically significant differences were most frequently observed for WC and WHtR, with girls presenting lower mean values than boys. Moreover, in the all-age sample, WC and WHtR significantly differed according to gender.

The prevalence of underweight, overweight, and obesity according to the BMI values were 4.9%, 22.3%, and 8.5%, respectively, without significant differences between the genders.

The proportion of the participants who had a high WHtR (>0.5) was 12.8%, and was higher in boys (14.3%) than girls (11.3%).

The WHtR age- and gender-specific percentile values are shown in Table 2.

**Table 2:** Waist-to-height ratio percentiles by age and gender

	Waist-to-height ratio percentiles								
	3rd	5th	10th	25th	50th	75th	90th	95th	97th
Boys age (y)									
6	0.40	0.41	0.42	0.44	0.45	0.48	0.50	0.52	0.53
7	0.40	0.41	0.42	0.44	0.46	0.49	0.52	0.54	0.55
8	0.40	0.40	0.41	0.42	0.45	0.48	0.53	0.55	0.56
9	0.39	0.39	0.40	0.42	0.44	0.47	0.52	0.55	0.55
10	0.39	0.40	0.41	0.42	0.45	0.48	0.51	0.54	0.55
11	0.38	0.39	0.40	0.43	0.47	0.50	0.53	0.57	0.58
Girls age (y)									
6	0.41	0.41	0.42	0.43	0.45	0.49	0.52	0.54	0.59
7	0.39	0.40	0.40	0.42	0.44	0.48	0.51	0.52	0.54
8	0.39	0.39	0.40	0.41	0.44	0.47	0.51	0.55	0.56
9	0.38	0.39	0.40	0.42	0.45	0.47	0.51	0.55	0.58
10	0.37	0.38	0.39	0.40	0.43	0.47	0.50	0.52	0.55
11	0.36	0.37	0.37	0.39	0.42	0.44	0.48	0.54	0.56

The classification according to the WHtR cut-off value of 0.5 was statistically associated with those obtained by using the BMI classes (Chi^2^: 810.16; d.f.: 3; *p* < 0.05).

The majority (96%) of children with a high WHtR were also overweight or obese as per BMI; 60% of the children ranked overweight or obese by BMI had a WHtR ≤0.5.

The correlation between WHtR and BMI was high for the overall sample (*r* = 0.766, *p* < 0.05) as well as on the basis of gender (boys: *r* = 0.803; *p* < 0.05; girls: *r* = 0.736; *p* < 0.05).

**Fig. 1:**
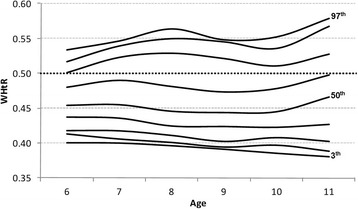
Waist-to-height ratio percentiles for boys

**Fig. 2:**
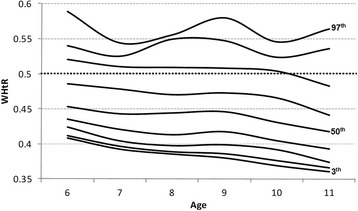
Waist-to-height ratio percentiles for girls

The WHtR percentile curves for boys and girls are depicted in Figs. 1 and 2, and the corresponding age- and gender-specific percentile values are given for the 3rd, 5th, 10th, 25th, 50th, 75th, 90th, 95th, and 97th percentiles in Table 2.

## Discussion

The prevalence of overweight and obesity in our sample was higher than the Tuscan sample: a research conducted in 2012 showed a regional prevalence of 19.6% and 7% for overweight and obesity, respectively, in both genders at 89 years of age [24]; the prevalence was similar to the national average values regarding overweight (22.3% vs. 22.2%) and lower regarding obesity (8.5% vs. 10.6%) [25].

With regard to WHtR, our results are similar to those of a Swiss study that analysed a large nationally representative sample of six- to 13-year-old children, and which reported identical WHtR mean values of 0.46 for boys and 0.45 for girls [11]. Age-specific Italian data are not available for a comparison at the national level, although some authors have described this parameter in a sample of obese children [26].

WHTR has several advantages over BMI in practice for identifying overweight and obese children in a population. First, it combines the advantages of both BMI and waist-to-hip ratio by taking not only height, but also the abdom-final adiposity into account. Secondly, it is less correlated with age than BMI, which makes it possible to propose a non-age-dependent cut-off point, as we did in our study, which is easy to manipulate for both professionals and lay people WHTR has several advantages over BMI in practice for identifying overweight and obese children in a population. First, it combines the advantages of both BMI and waist-to-hip ratio by taking not only height, but also the abdom-inal adiposity into account. Secondly, it is less correlated with age than BMI, which makes it possible to propose a non-age-dependent cut-off point, as we did in our study, which is easy to manipulate for both professionals and lay people. The relationship between anthropometric indexes of abdominal fat (waist circumference) and cardiovascular risk factors was also explored in Italy in both obese and non-obese children and adolescents at the beginning of this century [27, 28]. WHtR has several advantages over BMI in identifying high metabolic risk in children, even if some authors have stated that WHtR is useful in detecting a higher metabolic risk condition only in overweight children, and not in obese children, due to the high chance of cardiovascular risk factors positively associated with a high total fat mass [9, 26, 29–32]. There are mainly two advantages of WHtR over BMI: first, it combines the advantages of measuring BMI and WHtR since it considers height along with abdominal adiposity; and second, it is less correlated with age than BMI, which has led some authors to propose a non-age-dependent cut-off point that can be easily used by both professionals and lay people. Previous Italian studies also analysed the role of anthropometric indexes of abdominal fat (waist circumference), and found that they may be a good choice in clinical practice, in order to facilitate the detection of individuals with cardiovascular risk factors in childhood [27]. Moreover, they seem to be useful in order to identify sub-groups of already obese girls at higher metabolic risk [28].

With regard to percentile distribution, the 90th WHtR percentile crossed the 0.50 cut-off for both genders, in line with a Swiss study, where the 85th WHtR percentile lay exactly on the value of 0.50 across all age groups for boys and it crossed the 0.50 cut-off for girls [11]. In both cases, WHtR decreased slightly with the increase in age for girls. The highest percentiles (90th and 97th) for both genders showed a curvilinear profile, which was different from the results reported by other studies [11, 33]: this may represent an artefact due to the small sample size corresponding to these percentile values.

Data for 60% of children marked overweight or obese with BMI cut-offs but with a WHtR ≤0.5, similar to results obtained by Khoury et al., with 55% of the subjects categorized as overweight by BMI having a WHtR <0.5 [34].

The strength of our paper is that a consistent number of children were recruited and directly measured.

The main limitations arose due to sample recruitment—all the children were from a small geographical area (Tuscan)—which influenced the generalizability of the results; and to voluntary participation, which determined the lack of information on the non-respondent children (though in scarce percentage). Since we did not collect information about cardiometabolic risk factors, we could not discuss the use of the 0.5 cut-off value from a clinical point of view.

## Conclusions

WHtR is a simple, easy, inexpensive, highly reproducible, and accurate tool for prevention, control, and intervention against childhood obesity. Considering this, studies aimed to describe the distribution of these parameters in a large sample of Italian children, like the one described in this paper, could be useful for comparisons.

Given that many overweight and obese subjects had low WHtR, this measurement might be particularly useful in this population, and it can represent an important issue in terms of the need for public health assessments among the child population.

Owing to the limited geographical representativeness of our paper, further studies are needed to confirm our data at the regional and national levels. Moreover, larger scale prospective studies may be useful in order to better understand a possible effect of age on the WHtR value, so as to assess whether it is suitable to use a single cut-off point for detecting cardiometabolic risk, regardless of age, or different cut-offs for different classes of age.
